# ‘Out of sight, out of mind’ - results of a focus group study on gamblers’ use and evaluation of player protection measures in Germany

**DOI:** 10.1186/s12889-026-26892-6

**Published:** 2026-03-06

**Authors:** Anke Quack, Manfred E. Beutel, Lena Gieseke

**Affiliations:** 1 Competence Centre for Player Protection & Prevention (KSP), Department of Psychosomatic Medicine and Psychotherapy, University Medical Centre Mainz, Johannes Gutenberg-University Mainz, Untere Zahlbacher Str. 8, 55131 Mainz, Germany; 2 Department of Psychosomatic Medicine and Psychotherapy, University Medical Centre Mainz, Johannes Gutenberg-University Mainz, Untere Zahlbacher Str. 8, 55131 Mainz, Germany

**Keywords:** Gambling, Responsible gambling, Player protection, Consumer perspective, Prevention

## Abstract

**Background:**

The growing prevalence of gambling-related problems underscores the importance of effective player protection measures and highlights gambling addiction prevention as a critical public health challenge. However, the perspective of gamblers has so far rarely been taken into account in the development of player protection measures in Germany. Furthermore, research indicates that gamblers’ awareness and use of available information and support services for gambling problems are relatively low to moderate.

**Methods:**

To address this gap, a focus group study was conducted with a total of 19 gamblers to gather in-depth information into their views on player protection measures. Participants were screened for problem gambling behaviour using the Problem Gambling Severity Index prior to the discussions. Using a structured interview guide, participants were asked about their awareness and assessment of player protection measures as defined in the State Treaty on Gambling. They also evaluated various examples of both online and offline player protection information and suggested potential improvements. The interviews were recorded, transcribed and analysed through qualitative content analysis.

**Results:**

Information and messages on player protection were barely noticed and did not address the actual information needs of gamblers. Participants reported that their memories of responsible gambling messages were often vague and superficial, many were unaware of available counselling services. The critique highlighted issues such as poor visibility on websites, excessive text content and the preponderance of advertising vs. responsible gambling messages. The study identifies key improvements for responsible gambling (RG) communication: increasing RG visibility, using concise messaging, including social media and videos, implementing interactive tools and personalized feedback, and employing proactive warnings. RG content should be developed by independent organizations to increase credibility.

**Conclusion:**

Including gamblers in the development of player protection measures and messages, along with identifying their information needs, can contribute to the evidence-based discussion on player protection and enhance its effectiveness.

**Supplementary Information:**

The online version contains supplementary material available at 10.1186/s12889-026-26892-6.

## Background

The gambling market is growing worldwide. Online gambling has been legalized in many countries and is becoming the dominant form [1]. At the same time, gambling is increasingly recognized as a public health issue. A recent meta-analysis estimates that approximately 440 million adults worldwide engage in gambling at some level of risk, and around 72 million suffer from gambling disorder or problem gambling. The authors of the study emphasise that gambling is not just leisure but a harmful addictive behaviour posing growing public health risks in a digitalized world [2]. Although global trends point to a rising importance of online gambling, terrestrial gambling remains more popular than online forms in Germany, with the exception of online sports betting. The current German Gambling Survey provides detailed insights into the prevalence of risky and problem gambling behaviour. According to the survey, 6.9% of the population engage in high-risk gambling activities such as electronic gaming machines, casino games, sports betting, or KENO. Gambling Disorder was present in 2.4% of adults aged 18–70. Of these, 1.0% met criteria for a mild disorder, 0.7% for a moderate disorder, and 0.7% for a severe disorder. Vulnerability was particularly pronounced among young adults (18–25 years), 4.9% of whom met the diagnostic criteria for Gambling Disorder. Notably, despite the greater overall prevalence of terrestrial gambling, the largest proportion of individuals with a gambling disorder in the survey reported participation in online casino games, underscoring the high-risk potential of these formats [3]. These findings demonstrate that gambling constitutes a significant public health issue in Germany as well. The pronounced demographic disparities, particularly the disproportionately high prevalence among young adults, underscore the urgent need for targeted prevention efforts and appropriate regulatory measures.

Online gambling carries elevated risks due to its location-independent access, 24/7 availability, low entry barriers, anonymity, and continuous play with very short betting intervals, all of which increase susceptibility to excessive gambling and gambling-related harm [4, 5]. The high-risk potential of online gambling [6–9], which targets a younger, online-savvy audience, highlights the need for effective, evidence-based player protection measures.

In recent years, various measures have been introduced globally to mitigate excessive gambling and related harm, with responsible gambling (RG) as a key concept [10]. Most initiatives follow the informed choice model [11], emphasizing gamblers’ self-regulation. These include information on gambling risks, counselling services, player bans, and RG tools such as betting history and limit-setting options [12]. Additionally, behaviour monitoring tools (e.g., Playscan, Mentor) identify problem gambling patterns and provide personalized feedback [13]. Self-regulation strategies fall into two categories: behaviour change strategies to reduce or control gambling and protective strategies to prevent gambling-related harm [14].

The concept of responsible gambling has been criticized for shifting harm prevention responsibility onto individuals rather than gambling providers or regulators [15, 16]. Many measures, such as consumer education, have limited effectiveness in preventing addiction, while structural interventions like advertising bans and stricter regulations for high-risk gambling products remain underprioritized [10]. Studies indicate low usage of player protection measures and RG tools, with loss limits being the most common (23.2%), while other tools like self-exclusion and helplines are rarely used (0.8%–5.5%) [17]. In Australia, 83% of gamblers did not use any protection tools [18]. In another study, awareness of RG tools is high (65.8%–96.6%), but actual usage varies, with activity statements (88.4%) being the most utilised, followed by deposit limits (24.5%) and time-outs (8.1%) [1]. Several studies reported that problem gamblers tend to use RG tools more frequently than those with lower gambling risks [1, 17, 19].

Empirical findings on the effectiveness of RG tools are inconsistent [20]. Some studies indicate that personalized feedback can reduce gambling intensity among at-risk groups [21–25]. Several randomized controlled trials have also shown that warning pop-up messages and temporary self-exclusion tools can influence gambling cognitions and short-term behaviour, although overall effects tend to be modest [26, 27]. Experimental studies further demonstrate that wagering inducements, such as bonuses or free bets, can intensify gambling engagement and emotional responses, potentially undermining responsible gambling messages [28]. In addition, conceptual work highlights the importance of considering individual differences and gambling behaviour patterns when designing RG interventions, supporting calls for more tailored prevention strategies [1, 29–31]. An umbrella review, however, found little to no effect of RG tools overall, citing substantial gaps in the evidence base [29].

At the content level, studies have shown that general slogans, especially on signs in gambling venues, are largely ignored by gamblers [32–34]. The slogan ‘Gamble responsibly’, which is widely used in Australia, Canada and the United States, is well known, but is also largely ignored by gamblers and has hardly any influence on gambling behaviour [20, 35, 36]. The slogan ‘Take time to think’, which was newly introduced in the UK at the end of 2021, also showed no strong positive effects on gambling behaviour [20]. Moreover, a recent randomised online experimental study found that the often-used message “when the fun stops, stop” had no protective effect on actual gambling behaviour, and in one condition even resulted in higher gambling expenditure, pointing to a potential backfire effect of such generic slogans [37].

In Germany, gambling providers are legally required to implement player protection measures in accordance with Sect. 6 of the German State Treaty on Gambling (GlüStV 2021), which sets out requirements for social responsibility concepts (Sozialkonzepte). However, research on their impact remains limited [19, 38–42]. A study found high awareness of player protection measures, including warnings (82.7%) and counselling services (68.5%), with broad support for their legal implementation [30]. However, actual usage remains low, similar to international findings. Media sources (22.4%) are the primary information channels, while leaflets (7.6%), help and counselling services (0.8% -1.7%) are rarely used [19].

Engebø et al. [17] point out that gamblers’ attitudes influence their use of player protection measures, highlighting the need to consider their information needs when designing and implementing player protection measures [1, 17]. In Germany, consumer involvement in evaluating player protection measures is limited, except for studies on player bans [43, 44]. Furthermore, little is known about gamblers’ experiences with player protection measures and RG tools [13], underscoring the need for qualitative research to explore usage barriers and strategies to increase adoption.

### Present study

Although a range of player protection measures is available, their actual use among gamblers remains low. At the same time, little is known about gamblers’ attitudes towards these measures, their evaluation of existing responsible gambling tools, and their specific information needs. This lack of knowledge hinders the development of evidence-based gambling-related health information [45]. Against this background, three focus group discussions were conducted with gamblers of varying gambling intensities. The aim of the focus groups was to conduct an in-depth analysis of whether and how gamblers perceive and use information on gambling addiction and player protection, how they evaluate the content and messages, and what information needs exist. Using exemplary player protection measures, the discussions also explored suggestions for improving measures and messages related to player protection.

The study explores the following research questions: (1) What are participants’ thoughts and attitudes toward player protection and responsible gambling? (2) Which player protection measures and responsible gambling messages are they aware of? (3) How do they evaluate these measures and messages? (4) How would participants improve player protection measures and messages?

This research aims to support the development of research-based, target group-oriented player protection measures. Incorporating gamblers’ perspectives may help increase their reach and effectiveness.

## Methods

### Study design

Between March and June 2023, three focus group interviews were conducted at the Department of Psychosomatic Medicine and Psychotherapy, University Medical Centre Mainz. A facilitator led the discussions, supported by two research associates. Using an interview guide (see appendix) based on the research questions, participants were asked about their awareness and evaluation of player protection measures under the State Treaty on Gambling.

The interview guide covered three main topics: (1) General attitudes toward player protection, (2) Awareness, use, and evaluation of player protection measures, and (3) Assessment and potential improvements of exemplary player protection information (online and land-based). Participants could also introduce their own topics.

As part of the focus group procedure, participants first received a player protection leaflet from Lotto Brandenburg. Lottery products are the most widely used form of gambling in Germany [3] and the study received partial funding from Lotto Brandenburg. As the content and structure of player protection leaflets are largely comparable across the German federal states, the leaflet was used as a representative example of written player protection information. Participants were given approximately eight minutes to review the leaflet before providing feedback on its content, comprehensibility, and design, as well as suggesting potential improvements. To complement the evaluation of the leaflet, participants also reviewed online player protection information. The printed leaflet was provided by Lotto Brandenburg only. For the online evaluation, participants worked in pairs at a computer where the websites of Lotto Brandenburg and an additional state-licensed online gambling provider were pre-opened. The second provider was randomly selected from the official whitelist maintained by the Joint Gambling Authority of the Federal States (GGL). Participants were asked to locate and read the player protection information on both websites and to evaluate its visibility, accessibility, clarity, usefulness, and overall presentation, as well as to suggest possible improvements.

Because the content, structure, and placement of player protection information are broadly comparable across state lottery providers and other licensed online gambling operators in Germany, participants’ assessments can be considered transferable to similar materials.

Each session lasted an average of 115 min. Interviews were video-recorded (image and sound) and transcribed using f4transcript software.

### Participants and procedure

Study participants were recruited with a leaflet outlining the study’s purpose, procedure, and contact details. It was distributed at the Mainz outpatient clinic for gambling addiction, on the campus of the Johannes Gutenberg University Mainz, regional addiction counselling centres, and various gambling providers. Additionally, advertisements were placed on an online marketplace. Most participants (*n* = 5) were recruited via the advertisement placed on the online marketplace. A further four participants were reached through flyers distributed in a regional casino. The remaining participants were recruited in roughly equal numbers through local addiction counselling centres, patients of the outpatient clinic, and flyers posted on the university campus.

Inclusion criteria were age ≥ 18 (legal gambling age in Germany), German language proficiency, and gambling participation within the past 12 months. Participants received €20 for the preliminary interview and €50 for the focus group discussion. A preliminary interview was conducted before participating in the focus groups. This interview served to verify the inclusion criteria (legal age and gambling participation within the past 12 months). To confirm legal age, participants were asked to show the year of birth on an identification document, no additional personal information was recorded. The Problem Gambling Severity Index (PGSI) was also administered to assess the severity of problem gambling behaviour for descriptive purposes [46]. PGSI results were not automatically disclosed, but participants could request feedback if desired. The preliminary interview further provided an opportunity to inform participants about the study procedures and to address any questions. Interviews were conducted either online via VIOMEDI (a certified, data-protection-compliant telehealth platform) or in person at the university medical centre. The duration of the preliminary interview varied and depended on the participants’ individual questions regarding the study procedures.

A total of 19 gamblers participated (63.2% male, 36.8% female), with a mean age of 34.3 years (range: 19–63). Based on PGSI scores, 15.8% were non-problem gamblers (*n* = 3), 26.3% low-risk (*n* = 5), 36.8% moderate-risk (*n* = 7), and 21.1% problem gamblers (*n* = 4). Preferred gambling activities included slot machines (casinos/arcades), lottery products, online casinos, and sports betting (Table 1).

**Table 1:** Socio-demographic and gambling behaviour characteristics in each focus group (FG)

	FG1 (*n* = 6)	FG2 (*n* = 6)	FG3 (*n* = 7)	total (*n* = 19)
Sex				
female	4	0	3	7
male	2	6	4	12
Age				
mean age	35.7	26.2	40.1	34.3
range	20–62	19–33	25–63	19–63
Problem Gambling Severity Index				
Score of 0 = non-problem gambling	0	1	2	3
Score of 1–2 = low-risk gambling	2	1	2	5
Score of 3–7 = moderate-risk gambling	1	3	3	7
Score of 8 or more = problem gambling	3	1	0	4
Preferred form of gambling (multiple answers possible)				
Lottery products	3	1	5	9
Slot machines in casinos and gaming arcades	5	1	3	9
Online casinos	0	3	3	6
Sports betting	3	1	1	5

### Data analysis

The transcripts were analysed using qualitative content analysis following the approaches of Mayring and Kuckartz [47–49]. This method was selected because it provides a structured and transparent framework that accommodates both deductive and inductive category development. This was well suited to the aims of the study: deductive categories could be derived from the legally defined player protection measures specified in the German State Treaty on Gambling, while inductive coding enabled participants to introduce additional perspectives, needs, and evaluative criteria not captured by predefined regulatory concepts. The rule-guided nature of qualitative content analysis ensured consistent coding and facilitated systematic comparison across the focus groups.

Based on the interview guide and the research questions, a deductive category system comprising five main categories was developed: (1) perception of player protection, (2) awareness and evaluation of player protection measures, (3) access pathways and search strategies, (4) evaluation of exemplary player protection measures, and (5) suggestions for improvement. During the coding process, these main categories were complemented by inductively derived subcategories and dimensions, developed through open coding and comparative analysis of participants’ statements across multiple coding cycles. The further differentiation of the category system followed the analytical logic described by Mayring and Kuckartz and was guided by both the research questions and the content of participants’ contributions.

The transcripts were coded using the software f4analyse, which supported systematic data management and transparent documentation of analytic decisions. Relevant text segments were assigned to the deductive and inductive categories, and the coding system was iteratively refined to ensure adequate representation of the breadth and nuance of the data. Following this process, the results were summarised by category, descriptively analysed, and interpreted.

Coding was conducted collaboratively by the research team. Discrepancies were discussed until consensus was reached. Although no formal interrater reliability coefficients were calculated, the team-based approach, iterative coder discussions, and rule-based analytic procedures supported coding consistency. Nonetheless, the absence of statistical interrater metrics is acknowledged as a methodological limitation. To enhance analytic validity, the category system was also discussed in a peer-debriefing session with colleagues from the outpatient clinic for gambling addiction.

In total, 2,174 statements were coded and assigned to the main categories and subcategories.

The results were summarized, descriptively analysed, and interpreted. For publication, recurring themes were summarised and condensed. Numbers in parentheses indicate the number of coded statements within this category, which may exceed the number of participants because individuals could contribute multiple statements on a given aspect or specific question.

Representative participant quotes are included, with pseudonyms assigned based on PGSI score and group to ensure anonymity:

Non-Problem Gamblers (NProbG) + random number + group (1–3) (e.g., NProbG2, Group 3). Low Risk Gamblers (LRiskG) + random number + group (1–3) (e.g., LRiskG1, Group 2). Moderate-Risk Gamblers (MRiskG) + random number + group (1–3) (e.g., MRiskG2, Group 1). Problem Gamblers (ProbG) + random number + group (1–3) (e.g., ProbG1, Group 3).

This publication presents findings from the three key categories: ‘General attitudes towards player Protection’, ‘Awareness, use and evaluation of player protection measures’ and ‘Evaluation and optimisation of exemplary player protection measures (online and land-based)’.

## Results

### General attitudes towards player protection

At the beginning of the group discussions, participants were asked about their personal perceptions and attitudes towards player protection. During the coding process, key themes emerged regarding the tension between personal responsibility and state regulation and the perceived lack of credibility of gambling operators as providers of prevention measures.

#### Balancing personal responsibility and state regulation in gambling

Participants discussed the tension between individual responsibility and state regulation of gambling (13). Some questioned the extent to which gambling providers should be responsible for preventing gambling-related harm. They argued that individuals should take responsibility for their own gambling behaviour and criticised state regulations as excessive, restricting personal freedoms by mandating RG measures (8).

*‘The gambling provider is only responsible for ensuring that this information is available at the venue. I don’t think that the materials have to appeal to me. I still think that I am respon*sible for my gambling behaviour.’ (MRiskG3, Group 3).

*‘The other side is*,* of course*,* to what extent should the state intervene and regulate my personal space at all?*’ (LRiskG2, Group 1).

Conversely, other participants criticised the responsible gambling approach for placing too much emphasis on individual responsibility (5). They viewed self-regulation as particularly ineffective for those with gambling problems:

*‘You have to take the initiative yourself. You might be able to do that when you are in a stable state*,* but when the addiction takes over*,* that stability is lost. At that point*,* it becomes difficult to say on your own*,* “I won’t gamble now”.’* (LRiskG2, Group 3).

#### Credibility issues of gambling providers as initiators of gambling harm prevention

The credibility of gambling providers as initiators of gambling harm prevention was highly contested. Most participants viewed them as untrustworthy in this role (36), primarily due to a perceived conflict of interest. They questioned whether player protection could genuinely be a priority for operators:

*‘I find player protection by the operators controversial and somehow incompatible. On the one hand*,* you have a product that you sell*,* and on the other hand*,* you try to keep people from it. It doesn’t work.’* (NProbG1, Group 2).

Some participants believed that gambling providers implement player protection measures solely due to legal obligations (9):

*‘The gambling operators do it because they have to. By law. Full stop. Leave out the law and you can count on one hand how many gambling providers still do it.’* (LRiskG2, Group 3).

Additionally, concerns were raised that player protection measures are deliberately designed and placed ineffectively to avoid discouraging gambling, as this would conflict with operators’ financial interests (18):

*‘[As a provider] I wouldn’t saw off the branch I’m sitting on. I’d be stupid to implement player protection measures and suggest to [those affected] that they stop gambling immediately. As a provider*,* I want to make money from it.’* (ProbG1, Group 2).

#### Stronger government regulations on player protection measures

In contrast to participants who viewed state regulation as excessive and emphasised personal responsibility, some participants called for standardised government regulations on player protection (16), including legal requirements for safer gambling messages:

*‘It should be a legal requirement to display safer gambling messages to gamblers before*,* during or after gambling*,* whether online or offline.*’ (MRiskG3, Group 2).

Legal regulation should also cover the design of player protection measures:

*‘The design of player protection measures is not regulated. There are no rules on how they have to be presented to the customer. But there should be rules for them to be really visible. This must be clearly defined*,* otherwise the measures will miss the mark.’ (NProbG1*,* Group 2)*.

*‘The design of player protection measures should not be left to the principle of voluntariness. Either you involve an independent body or you make explicit specifications.’ (MRiskG3*,* Group 2)*.

### Awareness, use and evaluation of player protection measures

In the focus group discussion, participants were asked about their awareness, use, and evaluation of player protection measures and RG tools in accordance with the State Treaty on Gambling. Overall, they could name only a few player protection measures.

Analysis of statement frequency led to the categorisation of seven subcategories (inductive approach): (1) Warning messages: refer to the mandatory gambling-related notices (age restriction, addiction warning, help services) displayed on electronic gaming machines, tickets, advertisements, and gambling websites; (2) Information on help and counselling services: includes references to hotlines and counselling centres for individuals seeking support; (3) Player bans: include voluntary and operator-imposed exclusions from gambling venues or online platforms; (4) Staff intervention/feedback: describes situations in which staff members approach players or provide feedback on observed behaviour; (5) Limits: refer to monetary or time-based restrictions that players can set to control their gambling activity; (6) Information materials: include brochures, leaflets, and other written guidance on responsible gambling, also available on online gambling websites; (7) Win/loss statements: provide players with summaries of their gambling activity and financial outcomes.

Participants struggled to spontaneously recall or describe player protection measures in detail. However, warning messages (21), staff intervention/feedback (21), and help/counselling services (17) were the most frequently referenced. Key themes and opinions within these subcategories are summarized below.

#### Player protection information largely overlooked

Participants primarily mentioned stickers on vending machines and warnings at the end of media adverts. Many criticized their lack of visibility due to inconspicuous design (16). Stickers were often too small and blended into the background:

*‘On a black vending machine*,* a dark sticker with dark grey lettering is hardly noticeable.’* (MRiskG2, Group 3).

Warning notices were also frequently ignored, especially during gambling:

*‘I don’t read them. The print is too small*,* the casino is dark*,* and when I’m gambling*,* I won’t focus on a tiny piece of paper.’* (LRiskG1, Group 1).

Problem gamblers were particularly unlikely to notice warnings:

*‘When chasing losses or celebrating wins*,* you ignore warnings. It’s not like a huge banner suddenly pops up that you have to click five times*,* saying ‘addictive potential.’ The reality is*,* you simply don’t notice it in that moment. Only when I actively look for it do I find it.’ (*ProbG1, Group 2).

Few participants could name specific player protection measures in gambling venues (9). Some criticized that information was typically buried in the small print:

*‘Whether in adverts or leaflets*,* it’s always in the small print.’* (ProbG2, Group 1).

Most participants who had seen player protection leaflets or messages admitted they had not read them in detail. Their recollection of the content was vague (14):

*‘There might be something on the counter at the venue*,* but it’s not noticeable. I haven’t really looked at it or actively read through it.’* (LRiskG1, Group 2).

*‘In the lottery shop*,* there’s also the big notice board with very small lettering. I can’t say exactly what it says*,* but I think it goes in the direction of player protection. But I think that very few people actually read the whole text.’* (MRiskG1, Group 3).

Online gamblers occasionally mentioned the betting and transaction history (6) but found it easy to ignore:

*‘When you log in*,* the first thing that comes up is a “Your total bets and losses” window*,* which is progress. But this window is very small. Of course*,* you can click it away very quickly and then just start playing - out of sight*,* out of mind.’* (MRiskG3, Group 2).

#### Imbalance between gambling advertising and player protection

Participants criticized the imbalance between aggressive gambling advertising and subdued player protection messages (25). Promotional content is visually dominant, often overshadowing protection measures:

*‘The advertising is more present than the prevention measure. You see a big red button that says “Three free spins” and then you click on these three free spins. There might be a small button next to it that doesn’t flash and it might say something about player protection measures. It’s often a visual overload on the relevant websites.’* (LRiskG2, Group 3).

This disparity is also evident in media advertising, especially on the radio:

*‘Radio adverts hype jackpot millions*,* with sound effects and excited voices*,* and then at the very end comes “gambling can be addictive”*,* that’s how dryly it’s presented. And usually very quickly*,* because it takes up airtime.’* (MRiskG3, Group 2).

*‘In lottery radio adverts*,* they say very briefly “Gambling can be addictive. You can get information from XY.” But that’s two seconds. The advert lasts 20 seconds. The warning is very short.’* (NProbG1, Group 3).

#### Lack of staff feedback on gambling behaviour

Statements about staff intervention in gambling behaviour were frequently coded (21), yet few participants (4) were aware of employees’ player protection duties:

*‘I believe staff are required to address problem gambling behaviour at slot machines in the venue.’* (MRiskG3, Group 2).

The role of player protection officers was largely unknown:

*‘I’ve never heard of it in the casino. I think it’s great (…)*,* but I’ve never seen it before.’ (LRiskG1*,* Group 1)*.

When asked if they had ever been approached about their gambling behaviour or received online feedback, all participants denied it. One remarked:

*‘Well*,* it’s never really happened to me that someone has approached me personally and somehow worried that I should be careful or something. When I enter with friends*,* staff greet us warmly. At the bar*,* they already know our drinks - it feels welcoming.’* (MRiskG1, Group 1).

#### Limited awareness of help and counselling services

A total of 17 statements addressed the lack of awareness regarding help and counselling ser-vices. Most participants could not recall specific offers of help, particularly those offered by the BZgA (Federal Centre for Health Education, under jurisdiction of the Federal Ministry of Health in Germany). Despite providing information, materials, a website, and a gambling addiction hotline, the BZgA’s services remained largely unknown.

Although gambling adverts legally include references to counselling service, participants only remembered the general warning “Gambling can be addictive”, not the specific help re-sources:

*‘The phrase stays somewhere in your head*,* but on which site you can look it up already doesn’t.’* (MRiskG1, Group 3).

*‘I think there is information at the Federal Centre for blah blah blah … Education. But I can’t quite get it together. I think it’s the BZgA. I can’t remember the exact context in which I heard it*,* definitely in the context of gambling. But I can’t remember whether it was said in radio adverts or whether it was somewhere on the [online gambling provider] pages or in the [online gambling provider] adverts. But that’s dangerous half-knowledge.’* (NProbG1, Group 3).

Most participants were unaware of the BZgA’s telephone counselling service when asked directly:

*‘I can’t think of an official name. That’s a problem - something like this should be as well-known as an emergency number.’* (MRiskG2, Group 2).

Some attributed this to the lack of public visibility and advertising of the service.

Counselling and treatment services were rarely mentioned (3), and only one participant had sought help:

*‘The self-help groups I know are self-funded and run by former gamblers*,* not trained professionals. Finding and attending them is difficult.’* (ProbG1, Group 1).

#### Limit-setting requires personal initiative

Thirteen statements addressed limit-setting and in-game restrictions (e.g., play breaks). Several participants criticized that setting limits demands personal initiative, which is challenging for those with gambling problems:

*‘You can set a deposit limit*,* but it takes effort. You have to enter the minimum stake yourself - it’s not predefined.’* (LRiskG2, Group 3).

Some questioned the effectiveness of these measures (5):

*‘At my online provider*,* you’re banned for five minutes after an hour. But in that time*,* you just go to the toilet or grab something to eat.’* (MRiskG2, Group 3).

#### Player ban as an effective protection measure

Online gamblers in particular recognized the 24-hour ban as a useful tool for immediate self-exclusion and short-term protection (9):

*‘There is this measure*,* it’s like an emergency brake*,* you can use it to stop yourself for 24 hours. In the worst-case scenario*,* when you hardly have any control*,* you can exclude yourself for a short time.’* (MRiskG2, Group 2).

However, some criticized its limited duration, arguing it is insufficient to regain control or seek help. Participants familiar with the nationwide ban system OASIS^1^ rated it positively (7):

*‘If I self-exclude*,* and I actually feel that’s a small step forward*,* it’s immediately forwarded to OASIS […] and I’m also deactivated immediately. And then I am blocked for at least 12 months and after these 12 months I have to apply in writing to the Darmstadt Regional Administrative Council to be unblocked again. So the mechanism is not bad at all.’* (ProbG1, Group 2).

Some participants (6) advocated for stricter requirements to lift a gambling ban:

*‘If you’re banned*,* it means there’s a problem. There should be a higher hurdle to lift the ban - maybe a psychologist’s certificate confirming you’re stable*,* or the opposite: “No*,* you’re not allowed to play anymore.”’* (ProbG3, Group 1).

Particularly after a 24-hour ban, some participants emphasized the need for an active decision to resume gambling rather than automatic reactivation (4):

*‘I miss the inhibition threshold of having to lift the ban myself. Right now*,* it expires after 24 hours. Instead*,* I should have to actively decide - maybe click through a few steps or watch an educational video. And then I have to explicitly make this decision. Here*,* the decision is taken away from me.’* (ProbG3, Group 1).

### Evaluation of player protection measures: criticism & suggestions for improvement

In the focus group discussions, participants reviewed player protection materials, including:

- A prototypical leaflet from a German state lottery provider.
- Online player protection information from a lottery and an online gambling provider.

They assessed content and design and provided suggestions for improvement. The analysis identified key areas of criticism and recommendations that emerged during coding.

#### Optimizing player protection information: short, concise & interactive

After reading the leaflet and websites, Participants struggled to recall player protection messages, recommendations for action, or support services for problem gamblers (12):

*‘There are no recommendations for action.’* (ProbG1, Group 1).

*‘You don’t see anything about the actual measures*,* how it’s all supposed to work*,* how the customer is supposed to be specifically protected.’* (MRiskG3, Group 2).

Excessive text was a key criticism (33), hindering readability and discouraging engagement with the content:

*‘There’s far too much text. Nobody reads it.’* (LRiskG1, Group 3).

*‘I also noticed it straight away when reading: too long sentences*,* too much text. You lose interest in reading through it.’* (MRiskG2, Group 3).

Some participants criticized the absence of interactive features on gambling providers’ player protection websites, suggesting gamified content like self-tests (10):

*‘I like interactive offers. Since I like playing*,* I’d also engage with player protection in a playful way.’* (MRiskG2, Group 3).

#### Utilising new media for player protection communication

Integrating new media to address problem gambling characteristics, provide help, and promote responsible gambling can enhance the appeal of player protection information (8):

*‘Nowadays*,* everything is explained in videos - why not use them for player protection? You have one click and then you can address and thematise everything in the video*,* perhaps from a more neutral perspective. This could also come from a prevention website that is responsible for this. They could create a video to ensure the information comes from a neutral source rather than the gambling provider. I think more people would watch a video than read a text.’* (MRiskG1, Group 2).

Especially young people can be reached more effectively through social media (8):

*‘For all the younger ones*,* you could create short videos on Instagram or TikTok that explain problem gambling behaviour in just a few seconds or encourage reflection. There’s a lot you can do.‘* (MRiskG3, Group 2).

Influencers, YouTubers, and streamers are role models for many children and young people. The panellists suggested that they should use their role model function to draw attention to the issues of gambling addiction and responsible gambling:

*‘You really have to start with the kids […]. YouTubers and influencers have a lot of power. And the fact that a lot of young people have also started gambling is partly because really big YouTubers and streamers showed up and said: “Great*,* I bet €2 here and won €8*,*000*,* I’m in.” But then they don’t show that they’ve already gambled away 10*,*000 euros. It is precisely these people who have the power*,* who should sensitise young people and say: “Hey*,* talk more about it.” Some are now admitting*,* “I was addicted*,* I made mistakes.” I like that - it shows change*,* and maybe it influences young people*,* too.’* (MRiskG2, Group 3).

#### Avoiding counterproductive player protection messages

Some panellists criticised wording that trivialised the potential dangers of gambling (27):

*‘Such words as “gambling is a harmless leisure activity” are very expressive words that do not actually correspond to addiction prevention.’* (ProbG1, Group 2).

*‘It says something about thrills and feelings of happiness*,* entertaining*,* social*,* memorable pastimes. That’s just the wrong message.’* (LRiskG1, Group 1).

Statements like ‘Playing the lottery is hardly addictive for anyone’ or ‘Most people are able to control their gambling behaviour’ gave the study participants the impression that they were one of the few people affected:

*‘That sentence doesn’t help me to admit my addiction. I’m one of the very few*,* so I want it even less.’* (ProbG3, Group).

Furthermore, it was expressed comparatively frequently (15) that gambling addiction should be discussed more as a serious disorder to reduce stigma:

*‘You have to talk about gambling addiction without the person being judged. If I’ve had an appendectomy*,* I can also say “I’ve had an appendectomy”. In the same way*,* I should be able to say “I have a problem with gambling.” And then without prejudice*,* please […].’* (LRiskG1, Group 3).

*‘My conclusion for player protection measures: It needs to become more normalised. I would like to see less stigmatisation and easier access to information. I need to be able to access it easily*,* I need to be less afraid of coming out and it should simply be low-threshold and easy to access.’* (MRiskG3, Group 3).

Some participants suggested complementing the warning “Gambling can be addictive” with positive and encouraging messages (4) that highlight the treatability of gambling addiction.

*‘For example*,* the sentence “Gambling can be addictive”*,* perhaps adding “But you can also take action against it.” or adding what you can do against it.’* (MRiskG1, Group 3).

*‘I would like them to say “Addiction is not a weakness” or “Addiction is not a sign of a weak personality”. […] That there is no shame in seeking help.’* (MRiskG3, Group 3).

#### Stronger communication of gambling risks and player protection benefits

Participants called for stronger messaging on gambling risks and responsible play, as well as the benefits of player protection measures (21):

*‘You should always link an action to its impact. As a gambler*,* I want to know the benefits of the player ban. What do I get out of it? Why do I benefit?’* (ProbG1, Group 1).

*‘The incentive to gamble is emphasised in all materials*,* but there is no incentive stop. None at all.’* (LRiskG2, Group 1).

In the self-test in particular, the participants called for clearer action-oriented recommendations, especially for those answering “yes” multiple times (5):

*‘If I tick “yes” here*,* there is already the potential for an addiction problem. I should actually be given the clear advice “Contact an addiction counselling centre”. But it only says something like “Then you should change your gambling behaviour” […].’* (ProbG1, Group 2).

Regarding player bans, some panellists highlighted the need for greater visibility and direct links to counselling services:

*‘What I would improve? The presence. On the websites*,* the self-exclusion button or 24-hour stop should be permanently visible on the page*,* even before logging in.’* (LRiskG1, Group 2).

*‘If I’m excluded by the player ban*,* then perhaps it would also make sense to refer the person to addiction counselling or therapy […].’* (ProbG3, Group 1).

#### Improving visibility of player protection information

Panellists emphasized the need for clearer, more prominent player protection information in both land-based and online gambling (49):

*‘I think that the relevant information should be placed differently*,* both on the providers’ websites and at the entrances to casinos or lottery shops.’* (LRiskG2, Group 3).

*‘Prevention should be ‘advertised’ as much as bonus games. That would be a start.’* (MRiskG3, Group 2).

*‘If I google “gambling”*,* the number of the counselling hotline should come up immediately […]. If I have to click on 5 more websites and then into the submenu to find the number*,* I’ll give up […].’* (ProbG1, Group 2).

Participants frequently criticized the placement of player protection information at the bottom of gambling websites, perceiving it as intentionally hidden (49):

*‘I noticed that the information is well hidden. So much so that you could almost mistake it for the legal notice. If you just click through the page and scroll around a bit*,* you’ll end up at the bottom of the page without ever having paid attention to it. It’s done in such a way that it’s hidden so that it’s completely overlooked*,* even in terms of contrast. In principle*,* they really try to hide it.’* (NProbG1, Group 2).

As a key improvement, participants suggested making player protection information highly visible, such as a fixed menu item in the website header or a sticky menu that remains accessible while scrolling (35):

*‘Nobody scrolls all the way to the bottom. You need a direct link. When I open the page*,* it always catches my eye. There has to be a link right at the top*,* so to speak. Or a link that always moves with me when I scroll down the page. It always stays in the same place*,* but always travels with me and I can’t close it.’* (ProbG1, Group 2).

The use of images, bright colours and flashing elements would help to increase the perception of player protection information:

*‘If colourful elements pop up*,* they naturally catch your attention.’* (ProbG1, Group 1).

*‘Something has to flash or something if it’s already at the bottom.’* (ProbG2, Group 1).

Some suggested placing player protection messages before gambling offers or advertisements (4):

*‘It should actually be at the top where the jackpot is. That’s as far as you normally get when you’re on the site.’* (LRiskG1, group 1).

Further suggestions for improvement included mandatory, recurring warnings that cannot be skipped immediately (12):

*‘I would prefer it if all these messages were displayed again and again*,* whether you want them to or not. That no matter how much time you spend on the site*,* this notice just keeps popping up and you can’t click it away*,* like an unskippable YouTube ad. So that you really have to read through it […]. ’* (NProbG1, Group 2).

## Discussion

Gambling is increasingly recognized as a public health issue and poses a growing threat in today’s digitalized and globalized world [2], underscoring the need for effective, evidence-based player protection measures. Currently, the concept of responsible gambling is widely used to prevent or minimize excessive gambling and its associated harms [10].

However, focus group participants demonstrated limited awareness and recall of player protection measures. These were perceived as inconspicuous, small, and difficult to notice, resulting in vague and superficial recollections of information on gambling risks, responsible gambling tools, and available counselling services. This aligns with prior research reporting low utilisation of RG measures and limited engagement with player protection tools [1, 13, 17, 19, 42]. Newall et al. [50] investigated the effectiveness of warnings on slot machines and online platforms, concluding that current warnings employed by gambling operators are suboptimal. This is primarily due to the use of ‘sludge’ strategies, such as small font sizes and inconspicuous placement, which diminish visibility and hinder gamblers’ comprehension of player protection measures. Similar to the concerns raised by the focus group participants, Newall et al. emphasise the need for legally mandated standards regarding the wording, design, and placement of informational materials and warnings to ensure gamblers receive clear and effective communication.

When considering exemplary player protection measures, participants also criticized the excessive amount of text, noting that its presentation and wording trivialised gambling risks. To improve effectiveness, they suggested increasing the prominence of information, using a supportive tone, and emphasising the benefits of responsible gambling tools. Additionally, they recommended integrating interactive features to improve user engagement and effectiveness. Research supports these recommendations, showing that clear, non-stigmatising messages and interactive content enhance engagement [51–53].

Notably, no participants recalled being approached by gambling staff or receiving online feedback on their gambling behaviour, despite mandatory tracking tools in Germany. Various studies indicate that individual feedback on gambling behaviour can effectively encourage changes in gambling behaviour [21, 22, 24]. This raises the question of how feedback on gambling behaviour can be designed and presented to ensure it is noticed. One possible reason for the low visibility of personalized feedback is that gamblers must actively log into a designated personal information area to access their messages. Forsström et al. found that many users disengage from RG tools shortly after joining, suggesting that a more proactive dissemination strategy is needed [13, 16]. Repeated exposure to RG messages and dynamic, self-assessment-based formats may enhance effectiveness [24, 54].

Regarding the content of RG messages, study participants criticized the lack of specific calls to action. This aligns with the finding that gamblers respond well to specific loss warnings and actionable advice in RG messages [55]. Additionally, emotionally engaging messages, particularly those with personal relevance, are more effective in capturing attention and guiding gamblers towards responsible gambling behaviour by influencing their decision-making process [20, 54, 56]. To further enhance engagement with RG tools, it is essential to develop messages tailored to specific gambler groups (e.g., young adults or problem gamblers) and incorporate personalized feedback [13, 24, 51, 53].

Participants also criticized the disparity between the visibility of player protection messages and product advertising, noting that the latter predominates, often overshadowing responsible gambling messages and promoting gambling behaviour. This observation is consistent with previous critiques suggesting that player protection messages are frequently overpowered by highly salient advertising and promotional strategies [28]. Similarly, in a Finnish focus group study, the participants expressed concerns that gambling ads normalise gambling and may harm vulnerable groups, suggesting restrictions similar to those on alcohol and tobacco advertising [57]. Additionally, an eye-tracking study in Australia found that “gamble responsibly” messages in ads were rarely noticed by regular sports bettors due to their small size and poor placement [36]. These findings highlight the need for gambling advertisements to give greater priority to risk communication and ensure that responsible gambling messages are as visible as the advertisements themselves.

Building on the potential of digital communication, participants advocated for the use of new media to enhance RG messages, suggesting short, informative videos as an engaging way to convey information on problem gambling. They emphasised that videos produced by independent prevention websites would be perceived as more neutral and trustworthy than those created by gambling providers. Social media platforms were identified as particularly effective in reaching younger audiences. The strong emphasis on this topic may be attributed to the relatively low average age of the participants. However, research in other areas of health communication also indicates that younger individuals and frequent digital media users show high acceptance of prevention programs delivered via online platforms and social media [58, 59]. These media offer advantages such as easy accessibility and a degree of anonymity, which can be particularly beneficial for addressing stigmatised issues like mental health problems and addiction [60]. Studies further demonstrate that social networking sites and online interventions are effective in preventing alcohol and drug use [61–63]. Consumers tend to prefer well-designed and functional interventions accessible via smartphones, valuing the integration of professional feedback, gamified elements, and opportunities to share experiences with peers or former users [63, 64]. Delivering health-related information through new media may be particularly effective when incorporating interactive and personalised content to enhance user engagement.

The findings of this pilot focus group study suggest that, from the participants’ perspective, many existing player protection measures do not fully meet their informational needs. This aligns with the international scientific consensus, which calls for further research into the efficacy of individual RG tools [13, 51, 65]. In particular, it is essential to analyse the underlying motivations and barriers that influence the utilisation of these tools. Qualitative methods, for instance, could provide valuable insights into the reasons for adopting and abandoning RG tools [13].

Existing research and the findings of this study emphasise the need for future investigations to evaluate the effectiveness of different RG messages, taking into account linguistic and visual design elements as well as the specific needs of different groups of gamblers. Although initial impressions suggested possible differences between gamblers with different PGSI scores - particularly that participants with higher levels of problem gambling expressed stronger preferences for more prominent warning messages, including those incorporating fear appeals - these patterns were not confirmed in the qualitative analysis. For this reason, subgroup comparisons were not pursued further within this study. Instead, this question is being addressed in a subsequent quantitative follow-up study.

Understanding subgroup differences, such as those between younger and older gamblers or between recreational and problem gamblers, will be essential for developing more effective communication strategies. While highly personalised approaches are most feasible in online gambling environments, where continuous monitoring and screening tools enable targeted messaging, certain forms of tailored communication are also possible in terrestrial settings. For example, casinos and other land-based venues can offer age-specific information materials, integrate interactive formats, or use QR codes linking to risk-appropriate content. Although these approaches cannot achieve the real-time personalisation of online systems, they still provide meaningful opportunities to address the needs of different gambler subgroups and strengthen the effectiveness of player protection measures.

### Limitations

This study has certain limitations. Although data collection was conducted in a controlled group setting and followed a structured interview guide, the generalisability of the findings is constrained by the lack of sample representativeness and the use of a qualitative research approach. However, qualitative social research does not primarily aim to produce population-level generalisations, but rather to capture a diverse range of perspectives and experiences. In this study, the methodological approach enabled an in-depth exploration of gamblers’ individual viewpoints, yielding rich and nuanced insights into complex phenomena that are particularly relevant for the development of effective and audience-oriented player protection measures. In line with this aim, the study sought to capture a broad range of perspectives rather than a statistically representative sample. Thematic saturation was reached after three focus groups, as no new relevant themes emerged across groups. This sample size was determined a priori in accordance with methodological recommendations for focus group research, which suggest that two to three groups are typically sufficient to identify central themes within relatively homogeneous target populations [66]. Nonetheless, subgroup differences cannot be inferred from the present qualitative data and require further investigation. This issue is addressed in a subsequent quantitative follow-up study with a larger sample, allowing for a more systematic examination of potential differences among gambler subgroups.

It is important to acknowledge that the group setting may have influenced responses due to social desirability biases and group dynamics. While dominant participants may have shaped the prominence of certain topics, group interactions can also foster a ‘snowball effect’, whereby one participant’s input stimulates new and innovative ideas among others [67]. Additionally, the qualitative approach enabled participants to introduce perspectives and discuss topics that researchers had not initially anticipated [68].

Despite the limited representativeness and potential group influences, this study provides valuable insights for the practical implementation of player protection measures and the design of future research projects. Further research with larger and more diverse samples is needed to validate these findings and explore differences across various subgroups of gamblers.

### Conclusion and practical implications

This study is one of the first to explore German gamblers’ perspectives on player protection measures in accordance with the State Treaty on Gambling. It provides valuable insights into gamblers’ perceptions and preferences regarding effective communication of responsible gambling measures and messages, contributing to the development of player protection measures based on research findings.

The findings highlight several key implications for enhancing player protection measures:

1. Increasing the visibility of player protection information in both land-based and online gambling environments is essential to ensure that responsible gambling messages reach a wider audience.
2. Utilising concise language and digital media is particularly important for engaging younger gamblers. This includes integrating short, educational videos and utilising social media platforms for the dissemination of RG messages.
3. Developing interactive tools and offering personalised feedback can significantly enhance the effectiveness of responsible gambling interventions. It may be worth exploring to what extent messages can be tailored to specific subgroups of gamblers to enhance their impact.
4. Implementing proactive warning messages that reach all gamblers, irrespective of their gambling behaviour, can help raise awareness and encourage safer gambling practices.
5. Ensuring the credibility of responsible gambling content by having it developed and disseminated by independent organisations rather than gambling operators.

When designing and implementing player protection measures, scientific findings should be incorporated more extensively than before. Furthermore, regular evaluations of these measures are necessary to assess their effectiveness and respond appropriately to emerging challenges. In summary, while this study highlights existing deficiencies in responsible gambling communication in Germany, it also provides several recommendations for improvement.

## Supplementary Information

Supplementary Material 1.

## Data Availability

The anonymised transcripts of the focus group interviews can be made available from the corresponding author upon request. Code availability is not applicable.

## Footnotes

1 OASIS (Onlineabfrage Spielerstatus): Before gambling, the gambling provider checks whether the player is banned in OASIS.

## Abbreviations

BZgA: Federal centre for health education (Bundeszentrale für gesundheitliche Aufklärung, Germany)
FG: Focus group
GGL: Joint gambling authority of the federal states (Gemeinsame Glücksspielbehörde der Länder, Germany)
GlüStV: German state treaty on gambling (Glücksspielstaatsvertrag)
KSP: Competence centre for player protection & prevention (Kompetenzzentrum Spielerschutz & Prävention, Germany)
LRiskG: Low-risk gambler
MRiskG: Moderate-risk gambler
NProbG: Non-problem gambler
OASIS: National self-exclusion system (Onlineabfrage Spielerstatus, Germany)
PGSI: Problem gambling severity index
ProbG: Problem gambler
RG: Responsible gambling
