# Supplementary material for: ‘Out of sight, out of mind’ - results of a focus group study on gamblers’ use and evaluation of player protection measures in Germany
Source: BMC Public Health. 2026 Mar 6;26:1161. doi: 10.1186/s12889-026-26892-6 (PMC13063445; doi:10.1186/s12889-026-26892-6)
Supplement: Supplementary file 1 — Supplementary Material 1. [file 12889_2026_26892_MOESM1_ESM.pdf]

Interview guide: Focus group interview - gamblers' use and evaluation of player protection measures in Germany

Topic	Script
**Arrival of the participants**	[The participants gradually enter the room and are assigned to their seats]
**Welcome**	[Welcoming the participants and thanking them for their participation]<br><br>[Personal introduction of the facilitators]
**Brief introduction to the subject**	In Germany, the State Treaty on Gambling has been regulating gambling since 2008. This treaty legally obliges gambling providers to ensure player protection and to encourage gamblers to gamble responsibly, i.e. to prevent and fight the development of gambling addiction.<br><br>Against this background, gambling providers must implement various player protection measures, which we would like to discuss today.<br><br>Research findings show that the use of player protection measures by gambling participants is rather low.
**Aim of the focus group interview**	That is the reason why we are here today: We want to discuss with you whether you are aware of player protection measures, how you evaluate them and what you would improve about them.<br><br>There are no right or wrong answers or opinions. The aim of this discussion is to find out more about your personal opinions and views. Of course, personal

	views may differ within the group. This is very valuable for us!
**Procedure, group rules, confidentiality of data, recording**	**Information:** Before we start, we would like to give you some information about the process of this discussion<br><br>**Duration:** This focus group will last about 90 minutes<br><br>**Role of the facilitators**: We will not take part in the discussion ourselves, but will only ask the questions. In some cases, we may ask follow-up questions to make sure that we understand everything correctly. Our only aim today is to capture your personal views<br><br>**Rules:** We have thought about a few rules in advance so that we can make sure you all feel comfortable and that there are no negative influences on the discussion. I'll briefly outline the rules for you:<br><br>❖ **Confidentiality:** Everything we discuss here will of course be treated confidentially. We are only interested in the content of the discussion, not in who said what. In group discussions of this kind, it is common practice to record the discussion on video. The video makes it easier for us to focus on the discussion today and to analyze the discussion later. The video is only used for our documentation. I will now switch on the devices so that you can get used to being recorded.<br><br>**[Switch on devices]**<br><br>❖ **Discretion:** We should also agree that everything the participants say stays between us.<br><br>❖ **Audibility:** We ask you to speak loudly and clearly. It is also

	important that only one person speaks at a time - otherwise we will have difficulty understanding you in the video.<br><br>❖ **Not being disturbed:** Please switch your mobile phones to silent and flight mode for the duration of the discussion, or switch them off completely. It would be great if we were not interrupted.<br><br>**Introduction:** For data protection reasons, we will not conduct a round of introduction. Your real name will remain unknown to the other participants. You have therefore received a participant code from us, which consists of a participant number (1-6) and the group number (1,2,3).
**Starting the focus group discussion**	Do you still have any organizational questions? Then let us start our discussion now.
**Intro question**	Q1: In general, what are your thoughts on player protection and responsible gaming?
**Part 1: Awareness and use of player protection measures (unaided)**	Q2: We are very interested to know if you have ever noticed any information or messages about the potential dangers of gambling or how to gamble responsibly? And more specifically, have you ever used or looked for information or help services?<br><br>**[Collect answers]**<br><br>***Ask: Where, how, what, when?***<br><br>***Summarize: Did I understand that correctly?***<br><br>Q3: What messages or statements from the material do you remember?

	Ask: Do you have any further information in mind?<br><br>***Ask: Are there any other experiences?***<br><br>Q4: Have you ever received feedback on your gambling behavior? Either in person at the gambling establishment or, for example, as an e-mail in the context of online gambling participation?<br><br>***Follow-up questions:***<br><br>***What situation was this in?***<br><br>***Was this feedback helpful to you?***<br><br>Q5: We have now talked about your personal experiences in recent times. Now I would be very interested to know where and how you would search for information on gambling addiction and player protection. Where would you look? What information do you think player protection measures should contain so that you feel fully informed?<br><br>***Ask: What do the other participants think? What else should be included?***
**Transition to the next question**	If there are no more additions, we would like to ...
**Part II: Evaluation of exemplary player protection measures**<br><br><br>**Leaflet Lotto Brandenburg**	Now, we would like to go one step further by showing you examples of player protection information.<br><br><br>I am now handing out an information leaflet from Lotto Brandenburg, a state lottery company. You can find such a leaflet in this or a similar form in every lottery store as in casinos. Please take your time to look at the leaflet so that we can discuss it afterwards.<br><br>**[4 minutes time to look at the leaflet]**

	We have brought along a few specific questions.<br><br>Q6: What impression does this leaflet give you?<br><br>Q7: How relevant and interesting is this leaflet for you?<br><br>Q8: What recommendations and messages for responsible gambling do you notice?<br><br>Q9: How do you rate the comprehensibility and usefulness of the information and messages?<br><br>Q10: How do you feel about the wording and language? How would you like to be approached on this topic?<br><br>Q11: Can you give me 3 positive and 3 negative aspects of this information leaflet? No matter how small or important they are?<br><br>Q12: If you were responsible for this leaflet, what changes would you make immediately? How should such a leaflet be designed so that you would take it?<br><br>***Follow-up questions:***<br><br>***Are there any changes that you would make to the content/optics?***<br><br>***These leaflets are intended for all gamblers to pick up and read through, i.e. to provide general information. What do you think about that?***
**Online information on Responsible Gambling**	Now that we have looked at an information leaflet for lottery products, we would like to encourage you to take a look at information on the subject of gambling addiction and player protection from online gambling providers.

	To do this, we would first ask you to sit down in pairs at the computers provided.<br><br>First, we would like you to find and open the information on player protection on the **Lotto Brandenburg** website.<br><br>Then we would like you to find and open the information on player protection on the [online gambling provider] website that we have selected at random.<br><br>Please take a few minutes to read through the information on player protection at [gambling provider].de so that we can discuss it afterwards.<br><br>**[The participants have 6 minutes to find the relevant information]**<br><br>***Ask the participants to sit down at the table again, regardless of whether the information was found or not.***<br><br><u>Q13: How do you rate the findability of the information on gambling addiction and player protection?</u><br><br><u>Q14: What messages, recommendations or measures for responsible gambling did you notice?</u><br><br><u>Q15: How do you rate the comprehensibility and helpfulness of this information?</u><br><br><u>Q16: How do you feel about the wording and language?</u><br><br><u>Q17: Can you name 3 positive and 3 negative aspects of these websites? No matter how small or unimportant they are?</u><br><br><u>Q18: If you were responsible for this website, what changes would you make immediately?</u><br><br>***Follow-up question:***

	Are there any changes that you would make to the content/optics?<br><br>Q19: If you are considering participating in online gambling, or if you have already participated in online gambling, how would you rate the benefit of being informed about your bets, winnings and losses in the last 30 days before each game participation?<br><br>Q20: At registration, online gamblers are asked to enter a monthly deposit limit across all providers; the deposit limit may not exceed 1,000 euros per month. What do you think about that?
**Summary**	You have described your personal experiences and impressions in the context of player protection measures. We have also looked at examples. To conclude our focus group, we would like to ask you:<br><br>Q20: Was there any information or player protection measures that you felt was missing or that you would definitely add?<br><br>Q21: What would need to be improved or changed in player protection so that you would find these information interesting and relevant? How would you like to be approached on this topic? How should texts be worded and information designed, so that you would read them?<br><br>***Summarize suggestions for improvement***
**Outro**	We have now reached the end of the discussion.<br><br>Is there anything else you would like to share with us? What haven't we discussed yet, what else is important to you?

	Possibly answer questions<br><br>Great, thank you very much. If there is nothing else, we will now come to the end.<br><br>We would like to thank you very much for your participation. Your support is very valuable to us and today's discussion was very interesting and helpful.<br><br>If there is anything else you would like to share with us after today, please feel free to send us an e-mail.<br><br>[Say goodbye to the participants]
